# Plant-Derived Terpenoids: A Promising Tool in the Fight against Melanoma

**DOI:** 10.3390/cancers14030502

**Published:** 2022-01-20

**Authors:** Patrycja Kłos, Dariusz Chlubek

**Affiliations:** Department of Biochemistry and Medical Chemistry, Pomeranian Medical University in Szczecin, 70-111 Szczecin, Poland; dchlubek@pum.edu.pl

**Keywords:** melanoma, plant terpenoids, cytotoxicity, apoptosis, mitotoxicity, metastasis inhibition, MAP kinases inhibition

## Abstract

**Simple Summary:**

Despite the numerous therapies, melanoma remains the deadliest of all skin cancers; however, plant-derived terpenoids are defense molecules that have proven anti-cancer properties. In this review, we present the results of the search for anti-melanoma plant terpenoids. Additionally, we show the effects of combining terpenoids with standard drugs, radiation therapy, or other plant substances on melanoma cell lines and animal models. Finally, we present some examples of drug delivery systems that increase the uptake of terpenoids by melanoma tissue.

**Abstract:**

Melanoma is responsible for the highest number of skin cancer-caused deaths worldwide. Despite the numerous melanoma-treating options, the fight against it remains challenging, mainly due to its great heterogeneity and plasticity, as well as the high toxicity of standard drugs. Plant-derived terpenoids are a group of plant defense molecules that have been proven effective in killing many different types of cancer cells, both in in vitro experiments and in vivo models. In this review, we focus on recent results in the search for plant terpenoids with anti-melanoma activity. We also report on the synergistic action of combining terpenoids with other plant-derived substances, MAP kinase inhibitors, or radiation. Additionally, we present examples of terpenoid-loaded nanoparticle carriers as anti-melanoma agents that have increased permeation through the cancer tissue.

## 1. Introduction

### 1.1. Melanoma: Incidence, Staging, and Genetic Background

Melanoma is a malignant tumour originating from excessive, uncontrolled proliferation of melanocytes. Although it can develop in various body tissues, such as the uveal tract, leptomeninges and mucosal surfaces, its most common form is cutaneous [1] The main contributor to cutaneous melanomagenesis is UV radiation (UVR), which not only exerts its effect by inducing cell death, but also directly damaging skin cell DNA [2]. Two types of UV radiation are responsible: UVA (31–400 nm) and UVB (280–315 nm). The latter directly damages DNA causing the formation of photoproducts, such as cyclobutene pyrimidine dimers (CPDs), or 6-4 photoproducts (6-4PPs). Additionally, it contributes to melanoma progression by triggering macrophage and neutrophil infiltration into the skin, which subsequently promotes angiogenesis, as well as melanoma cell survival, invasion and metastasis. UVA, which is much more abundant in the sunlight than UVB, directly induces oxidative stress in melanocytes and promotes its accumulation, which in turn results in oxidative stress-induced DNA damage [3]. Accordingly, the highest incidence of melanoma is reported among fair-skinned Caucasian populations, due to low photoprotection associated with a low amount of melanin [4]. Adolescent and young adult women suffer from melanoma more often than men [5], which may be partly due to the more frequent use of sunbed tanning [6]; however, after age 40, the incidence of melanoma is higher in men [7].

Melanoma staging results from the clinical and histological assessment of patients suffering from this disease. The eighth edition of the TNM system (T—tumour, N—nodal, M—metastatic) proposed by the American Joint Committee on Cancer in 2018 remains the most widely accepted approach to the staging of this tumour. The system includes eight clinical stage groups (0, I (A, B), II (A, B, C), III, IV) which are distinguished by the absence or presence of nodular or distant metastases: where stage 0 means ‘melanoma in situ’; stages I and II refer to a localized disease; stage III indicates one or more lymph nodes with metastatic involvement but no distant metastases detected; and stage IV refers to melanoma with distant metastases. Additional characteristics are assigned to each stage group to allow for precise identification of the advancement of the disease. These characteristics include histological parameters describing the degree of local advancement (e.g., tumour thickness and the presence of ulceration), number of involved lymph nodes, location of distant metastases, and serum level of lactate dehydrogenase (LDH) [8].

**Figure 1:**
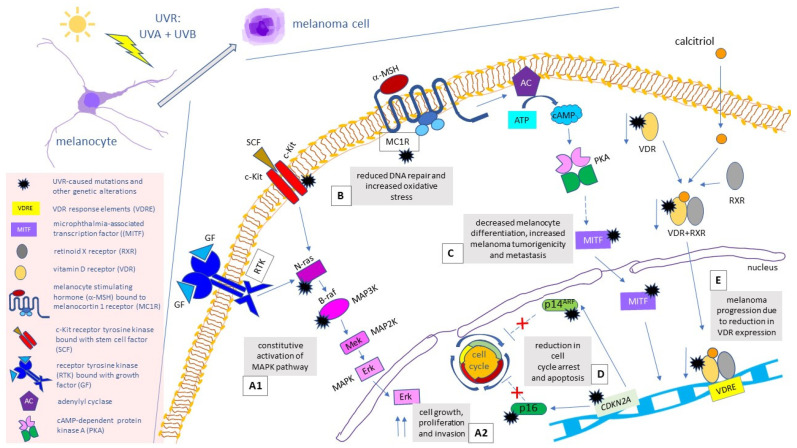
Mutations and polymorphisms associated with cutaneous melanoma and their effects for the development and progression of melanoma. (**A**) Mutations in B-raf (MAP3 kinase), N-ras, or c-Kit proteins result in the constitutive activation of MAPK pathway (B-raf → Mek → ERK) (**A1**), leading to intensified cell growth, proliferation and invasion (**A2**); (**B**) Genetic alterations in melanocortin 1 receptor (MC1R) may result in reduced DNA repair and increased oxidative stress; (**C**) Genetic alterations in microphthalmia-associated transcription factor (MITF) are associated with reduced melanocyte differentiation and increased melanoma tumorigenicity and metastasis; (**D**) Mutations in the *CDKN2A* gene coding for p16 and p14^ARF^ proteins result in a reduction in cell cycle arrest and decreased apoptosis; (**E**) Reduction in vitamin D receptor (VDR) expression affects melanoma behavior, contributing to the progression of the disease.

The onset of melanoma has been attributed to specific mutations in the signaling pathways that control crucial cell processes, such as growth, differentiation, migration, division and apoptosis, particularly in the mitogen-activated protein kinase (MAPK) pathway (Figure 1) [9]. Dysregulation of the MAPK pathway leads to cell cycle disturbance and uncontrolled cell growth. The MAPK pathway becomes activated upon binding a growth factor to a receptor tyrosine kinase (RTK) in a plasma membrane, which then leads to stimulation of the GTPase of a RAS protein. This, in turn, triggers the kinase cascade (MAP3K→MAP2K→MAPK), which activates transcription factors (e.g., c-Myc) and stimulates transcription of growth-related genes [10]. The most common mutation of the MAPK pathway concerns *BRAF*—a gene that codes for BRAF protein kinase, one of the MAP3 kinases. This genetic alteration occurs in 40–60% of cutaneous melanoma cases and results in a constitutive activation of the MAPK pathway [11,12]. The V600E mutation—a valine to glutamic acid substitution within the activation segment—is the most frequent genetic alteration in *BRAF*, accounting for 80% of all mutations in this gene. It is characteristic of younger people, skin that does not have cumulative solar damage (CSD) (i.e., extremities) and the superficial spreading subtype of melanoma [13]. The second-most frequent BRAF mutation—V600K (a valine to lysine substitution) is mainly encountered in the elderly and in CSD sites [14,15].

Other melanoma mutations associated with the MAPK pathway take place within *NRAS* and *KIT* genes and account for 15–30% and 1–2% of cutaneous melanoma genetic alterations, respectively [16,17]. Genetic alterations leading to constitutive, ligand-independent activation of c-Kit—a stem cell factor (SCF)-binding RTK [12,18]––are often encountered in mucosal and acral melanomas and in tumours arising from CSD-affected skin [19]. As for hereditary melanoma, its most common mutation, which is present in 40% of families with strong family history of this tumour, is the alteration in the *CDKN2A* gene, which codes for the proteins p16 and p14ARF, both of which acting as tumour suppressors [12,20]. Polymorphisms in the gene that codes for the vitamin D receptor (VDR) may also be relevant to melanoma development. Upon binding the active form of vitamin D (1,25-dihydroxycholecalciferol, or calcitriol), VDR creates a trimolecular complex with retinoid X receptor (RXR) and translocates it to the nucleus, where it binds to VDR response elements (VDRE) on DNA and acts as transcription factor. Loss of VDR expression has been correlated with melanoma progression [12,21]. Other melanoma-related genetic alterations consist of polymorphisms in genes coding for: melanocortin 1 receptor (MC1R) and melanocyte inducing transcription factor (MITF), also known as microphthalmia-associated transcription factor [22,23]. By binding the melanocyte stimulating hormone (MSH) to the surface of melanocytes, MC1R regulates skin melanogenesis and pigmentation [24] and enhances antioxidant defense [25] and DNA repair [26]. It has been described as a major gene involved in the development of sporadic melanoma [22], and its variants have been associated with the increased penetrance of *CDKN2A* mutations in melanoma-prone families [27]. The MITF stimulates melanogenesis by activating the expression of the tyrosinase and other pigmentation-related genes [28,29], and regulating melanocyte survival and proliferation [30,31]. It has been proven that the carriers of a germline missense substitution in this gene (Mi-E318K) had a more than fivefold higher risk of developing melanoma, compared to controls [29]. Figure 1 provides a graphical summary of mutations related to cutaneous melanoma and their results.

### 1.2. Melanoma: Currently Used Therapies

Although modern therapies for melanoma target a variety of mechanisms that enable the cancer to develop and survive, cutaneous melanoma is still responsible for 0.6% of cancer-caused deaths worldwide [32]. The choice of the method of treatment depends on the tumour’s location, stage, and the mutation underlying the development of the cancer. For localized cutaneous melanoma, excision with margins of healthy skin remains the best treatment option. Additionally, selective lymphadenectomy may be needed, in case of enlarged, palpable local lymph nodes [33]. Systemic treatment methods must be implemented if distant metastasis or an inoperable melanoma are diagnosed. Of them, immunotherapy alone, or in combination with MAP kinases’ inhibitors, is currently the standard treatment option [12].

The antibodies used in anti-melanoma immunotherapy target so called ‘immune checkpoints’: cytotoxic T-lymphocyte antigen-4 (CTLA-4) and programmed death-1 (PD-1), receptors on the surface of activated T cells. Upon binding to their ligands, the receptors attenuate T-cell response during the priming phase of immune response (CTLA-4), or in peripheral tissues (PD-1), thus preventing destructive inflammation. In turn, their blockade by antibodies augments the antitumour T-cell response [34]. So far, two anti-PD-1 antibodies (pembrolizumab and nivolumab) [35] and one anti-CTLA-4 antibody (ipilimumab) have been approved for melanoma treatment [36].

Regarding the MAPK inhibitors, the therapy combining BRAF and MEK inhibitors has proven its effectiveness in the treatment of V600 BRAF-mutated melanoma. Three combinations of these inhibitors (dabrafenib and trametinib [37], vemurafenib and cobimetinib [38], encorafenib and binimetinib [39]) have received the FDA approval for the treatment of unresectable settings. Additionally, the combination of dabrafenib and trametinib has also been approved as an adjuvant therapy post resection of stage III/IV melanoma [34].

Another treatment option for advanced-stage melanoma is the first FDA-approved oncolytic therapy—talimogene laherparepvec (T-VEC), based on the genetically modified herpes simplex virus, type I. The drug can be administered in the form of intratumoural injections for the local treatment of unresectable lesions in patients with recurrences after initial surgery [40]. Intralesional application of Mycobacterium bovis Bacillus Clamette-Guérin, an anti-melanoma vaccine, promotes T-cell responses and improves M2-macrophage function in patients with metastatic melanoma [41].

Standard tumour therapies, such as chemotherapy or radiotherapy, are implemented only in well-defined cases. Chemotherapy is not used routinely to treat melanoma due to a low treatment response rate [42]; however, in a form of isolated limb perfusion (ILP)/isolated limb infusion (ILI) combined with hyperthermia, it exerted its cytotoxic effect in a treatment of in-transit metastases [43]. Radiotherapy is used as local treatment in cases where radical surgery is not possible after an incomplete surgical resection of the tumour lesion, and as a palliative treatment in the case of brain metastasis [44].

Despite the numerous strategies for treating melanoma, the fight against the metastatic stage of the disease remains a challenge, mainly due to the tumor heterogeneity and plasticity, that is associated with the development of resistance to treatment [42]. New approaches are needed to enhance existing anti-melanoma therapies or develop a novel therapy.

## 2. Terpenoids as Anti-Melanoma Agents

### 2.1. Chemical Structure and Function of Terpenes/Terpenoids of Plant Origin

**Figure 2:**
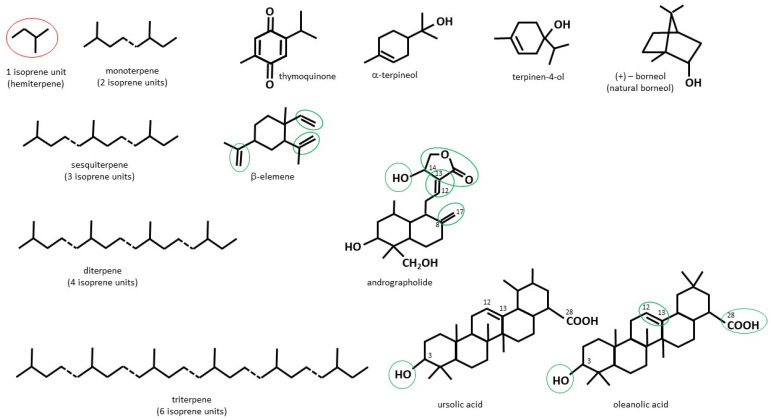
Chemical structures of each discussed terpene type (based on isoprene unit) and representative monoterpenoids (thymoquinone, α-terpineol, terpinene-4-ol, natural borneol), sesquiterpenoids (β-elemene), diterpenoids (andrographolide), and triterpenoids (ursolic and oleanolic acid). Green ovals and circles indicate structural elements that have been identified as the most important pharmacophores for the anti-cancer activity of the molecules.

Plant terpenes are a large group of structurally diverse compounds based on the five-carbon isoprenoid unit (IU) (Figure 2). Depending on the number of IU repetitions, they can be classified as: monoterpenes (2IU, or C10), sesquiterpenes (3IU, or C15), diterpenes (4IU, or C20), sesterpenes (C25), triterpenes (C30), tetraterpenes (C40) and polyterpenes (C > 40). Terpenoids are a subtype of terpenes that contain additional functional groups [45]. Although terpenes and terpenoids are not chemically identical, these two names are often used interchangeably [46]. In this review, ‘terpenoids’ will be used to refer to both groups of compounds.

Terpenoids perform a number of important functions. They act as signaling molecules, help plants response to sudden temperature changes, or ward off pathogens and competitors. By doing so, they protect plants from various abiotic and biotic stresses [47]. Smaller molecules, such as monoterpenoids or sesquiterpenoids are often found in essential oils, whereas the ones with larger molecular weight (e.g., triterpenoids) are mainly present in balsam and resin [45]. In medicine/naturopathic medicine, terpenoids are valued for their antibacterial, antiviral, antifungal, antiparasitic, anti-inflammatory, analgesic, and anti-cancer properties [46,48]. Paclitaxel, a terpenoid originally derived from the bark of the Pacific yew (*Taxus brevifolia*), is a chemotherapeutic widely used to treat a wide range of cancer types, including breast, ovarian, cervical, pancreatic, or esophageal cancer, and Kaposi’s sarcoma [49]. Insufficient efficacy in the treatment of metastatic forms of many cancers, including malignant melanoma, drives a constant search for new compounds with anti-cancer properties. The following review aims to present the results of the search for an effective substance from a group of plant terpenoids over the last eight years.

### 2.2. Monoterpenoids

#### 2.2.1. Thymoquinone

Thymoquinone (TQ) (Figure 2), a major component of the essential oil (EO) of black seed (*Nigella sativa*), is a quinone-based phytochemical showing keto-enol tautomerism. Of these two tautomeric forms, mainly the keto-form exhibits pharmacological activity [50]. In the organism, TQ can be enzymatically converted to thymohydroquinone or to semiquinone. The latter has been recognized as an oxidative stress-producing molecule in cancer cells. Although the superoxide anion can be nullified by TQ, the accumulating superoxide may force a pro-oxidant effect of TQ in tumour cells that lack detoxifying enzymes [51]. The low molecular weight of TQ (164.204 g/mol) facilitates its penetration across the blood–brain barrier, which makes it an attractive drug candidate from the anti-cancer drug development perspective for its potential use in treating brain tumours or brain metastases. Although lipophilicity and poor water solubility of TQ can limit its potential in the pharmaceutical industry, these can be overcome by incorporating this monoterpenoid into nanocarriers, thus increasing its bioavailability [50,52].

Thymoquinone has long been known in the scientific literature as a herbal active ingredient with antiviral, antibacterial, anti-inflammatory, anti-arthritic, antioxidant or ROS-enhancing (dose-dependent), and anti-cancer properties [53,54,55]. It has been proven effective against breast, lung, gastric, colon, prostate, ovarian, liver, cervical, head and neck, and skin cancers, as well as against leukemias [56]. In the study on murine malignant melanoma cell line, B16F10, Hatiboglu et al. showed that TQ reduced cell proliferation in a dose-dependent manner, with an IC_50_ value of 60 µM. Moreover, TQ induced the DNA damage and intracellular ROS generation, with the statistically significant effectiveness at the lowest concentration of 5 µM and 180 µM, respectively. TQ-induced apoptosis was shown to be induced by the blockage of Janus kinase 2 (JAK2)/signal transducer and activator of transcription 3 (STAT3) pathway and subsequent decrease in the expression of anti-apoptotic proteins (Bcl-2 and survivin), and an increase in the expression of the pro-apoptotic protein-Bax, and caspase 3. Additionally, TQ was found to decrease the expression of cytokines responsible for angiogenesis, immune suppression and tumour progression, such as monocyte chemoattractant protein-1 (MCP-1), transforming growth factor-β1 (TGF-β1), and RANTES (regulated on activation normal T cell expressed and secreted), in a dose-dependent manner. In an in vivo experiment, C57BL/6J mice with a metastasized intracerebral melanoma and treated with a daily TQ dose of 10 mg/kg of body mass showed a longer median overall survival time than the tumour-bearing control mice [57]. The same research group reported a longer median overall survival of intracerebral melanoma metastasis-bearing mice treated with a combination of TQ and gamma knife (stereotactic radiosurgery), compared to the control group of animals. However, the treatment with combination therapy had no advantage over the treatment with radiosurgery alone, presumably due to the limitation in the penetration of the blood–barrier by TQ, in the intracerebral melanoma model [58].

Similarly, Jeong et al. observed a reduction in the viability and proliferation of B16F10 murine melanoma cells after a 48 h treatment with TQ (5–30 μM). Thymoquinone at the concentration of 15 μM also decreased the expression of the regulators and participants of melanogenesis––β-catenin, microphthalmia-associated transcription factor (MITF) and tyrosinase––both at the protein and mRNA levels and reduced the activity of tyrosinase [59]. As shown by Slominski et al., stimulation of melanogenesis elevates the overall expression and increases nuclear localization of hypoxia-inducible factor-1α (HIF-1α), a subunit of HIF-1 transcription factor involved in regulation of glucose metabolism, angiogenesis, and stress responses. Moreover, the presence of elevated levels of HIF-1α in advanced melanomas may suggest its involvement in melanoma progression at local levels [60]. In this context, the inhibition of melanogenesis in melanoma cells appears to be important for increasing the effectiveness of melanoma treatment. To increase the uptake of TQ by the cancer tissue, Ibrahim et al. developed TQ-loaded poly(lactic-co-glycolic acid) nanoparticles (TQ-PLGA NPs) and tested its anti-cancer properties on A375 human melanoma cell line. The prepared TQ formulation expressed its dose-dependent cytotoxicity in A375 cells, with the highest cytotoxic effect registered at the concentration of 7.5 mg/mL, after a 48 h treatment. The IC_50_ value of TQ-PLGA NPs was between 2.5 and 5 mg/mL. However, the authors noted that the low stability of thymoquinone and the observed cytotoxicity of the suspension of blank nanoparticles, possibly due to their aggregation at higher concentrations, require further investigation [61].

Thymoquinone in combination with the callus extract of Iksan526, a resveratrol-enriched transgenic rice, was tested by the group of Eo et al., on A375 and B16F10 cell lines. The combination treatment proved to have enhanced anti-melanoma effect expressed as increased reduction in cell proliferation (A375 and B16F10), decreased expression of tyrosinase via the MAPK pathway (A375) and lowered expression of tyrosinase and cyclooxygenase-2 (COX-2) via the p38 kinase pathway (B16F10) [62].

#### 2.2.2. Terpineols

Terpineols (Figure 2), monocyclic monoterpene tertiary alcohols, are naturally occurring components of plant EOs. Of these, the most widespread in nature are α-terpineol and its isomer—terpinen-4-ol [63]. The first of the two compounds is found mostly in oregano, basil, and sweet flag, whereas the second one is mainly present in the EO of *Melaleuca alternifolia* (tea tree) [63,64,65]. The anti-tumour effects of terpineols have been tested on different cell lines, including lung, breast, colorectal, leukemia, pancreatic, prostate and gastric [66,67]; however, terpineols are also known for their antibacterial, anticonvulsant, antiulcer, antihypertensive and anti-nociceptive activity [63,65]. Alves Batista et al. assessed in silico the potentiality of α-terpineol’s becoming a drug. Among the number of identified properties, those that could contribute to its anti-cancer activity were particularly interested. These were: high permeability through the blood–brain barrier and high versatility of possible molecular interactions with classes of ionic receptors, membrane receptors, transcription factors, transporter molecules and enzymes. The authors synthesized poly(methyl methacrylate) (PMMA) nanoparticles containing α-terpineol and tested its cytotoxicity against melanoma on murine (B16F10) and human (SK-MEL-28) cell lines. The study showed that the α-terpineol/PMMA nanoparticles in a concentration of 5, 50 and 500 mg/mL reduced cell viability in B16F10 cell line by ca. 41, 54 and 56%, respectively, during a 48 h incubation. Similarly, the viability of SK-MEL-28 was reduced by ca. 54 and 59%, when 50 and 500 μg/mL concentrations of the nanoparticles were used. At the same time, α-terpineol/PMMA proved to be nontoxic to murine macrophages and human fibroblasts (MRC-5) [68].

The significant dose-dependent decrease in cell proliferation/viability was observed in human melanoma cell lines (M14 and A375) after their 48 h treatment with terpinen-4-ol, as reported by Di Martile et al. The authors observed the cytotoxic effect of the compound already at the concentration of 7.4 μg/mL. Interestingly, the pre-treatment of both melanoma cell lines with terpinen-4-ol synergistically decreased cell viability when followed by dabrafenib or trametinib administration. The combinatorial effect of the tested compound and the anti-melanoma drugs was proved to be associated with the induction of apoptosis, which was observed as increased poly(ADP-ribose) polymerase (PARP) and caspase 3 cleavage. According to the authors, the synergistic effect of terpinen-4-ol with standard therapeutics might be due to its lipophilic character and ability to interact with phospholipids of plasma membranes, which might result in reorganization of their lipid architecture and easier entry of the drug into the cell [69].

#### 2.2.3. Borneol and Its Esters

Borneol (Figure 2) is a bicyclic monoterpenoid that can be found in *Valeriana officinalis*, *Dryobalanops aromatica Gaertn f.*, or *Blumea balsamifera DC*. Its observed anti-cancer effect is to induce apoptosis and increase the permeability of the blood–brain barrier, which may facilitate transposition of drugs [70,71]. It has also been proved that it can enhance the effects of standard chemotherapeutic agents [72]. In turn, some borneol esters have been found to exert a pro-apoptotic effect [73]. Chen et al. investigated the apoptosis-inducing effects of the combination of natural borneol (NB) and curcumin in human melanoma cells. In the study, A375 cells were pretreated with NB (40 μg/mL) for 12 h, followed by the administration of different concentrations of curcumin, for 72 h. The authors demonstrated that borneol effectively enhanced the uptake of curcumin by A375 cells, which resulted in the activation of both extrinsic and intrinsic apoptotic pathways observed as cleavage of caspases 3, 8, and 9 and PARP, as well as DNA fragmentation and nuclear condensation. The mechanism of apoptosis was shown to be associated with increased ROS generation, enhanced expression of p53, ataxia–telangiectasia mutated (ATM), and Brca1, decreased phosphorylation of Akt and ERK1/2, and up-regulation of phosphorylated c-Jun N-terminal kinase (JNK) [74]. The effect of enhanced curcumin uptake by the cells pretreated with borneol could be due to the low molecular weight and high lipophilicity of this monoterpene. However, other mechanisms, such as loosening of tight junctions and the loss of mucous viscosity and elasticity have also been proposed to explain its drug penetration enhancing effect [75,76].

The anti-melanoma effect of betel-isolated bornyl *cis*-4-hydroxycinnamate (3, 6 and 12 µM) was studied by Yang et al. in two human melanoma cell lines (A2058 and A375). The treatment of the cells with the borneol ester resulted in a loss of mitochondrial transmembrane potential (∆ψm) and increased expression of Bad, Bax and cytosolic cytochrome c, in the both examined cell lines, suggesting the activation of mitochondria-mediated apoptosis. The involvement of an apoptotic pathway was further confirmed by the registered decreased levels of anti-apoptotic Bcl-2 and Bcl-xl, and elevated levels of cleaved caspases 3/9 and PARP. Moreover, ER stress pathway was found to be involved in bornyl *cis*-4-hydroxycinnamate-induced apoptosis, which was supported by the increased expression of protein kinase RNA-like endoplasmic reticulum kinase (PERK) pathway elements (PERK/αIF2a/ATF4/CHOP) [77].

The continuation of the study on bornyl *cis*-4-hydroxycinnamate by Yang et al. shed light on the mechanism by which the borneol ester acts on melanoma metastasis. The compound lowered cell migration in A2058 (at 1, 3, and 6 µM concentration) and in A375 (at 3 and 6 µM concentration) and inhibited the invasion of both cell lines (at 3 and 6 µM concentration). Moreover, it reduced the activities of matrix metalloproteinases MMP-2 and MMP-9, decreased phosphorylation of MAPK signaling pathway-related proteins, reduced the expression levels of the members of metastasis-related focal adhesion kinase (FAK) (FAK/PI3K/Akt/mTOR) and growth factor receptor protein 2 (GRB2) signaling pathways, in a dose-dependent manner. Finally, by decreasing the levels of N-cadherin and nuclear Snail and increasing the expression of E-cadherin, bornyl *cis*-4-hydroxycinnamate blocked epithelial-to-mesenchymal transition (EMT) in melanoma cells [78].

Similar research was performed by Wu et al., who tested the effect of betel-extracted (+)-bornyl *p*-coumarate (6, 12, 18, and 24 µM) on A2058 and A375 cells lines. The treatment of the cells with the borneol ester led to mitochondrial loss of function, up-regulation of the cleaved caspases 3/9 and PARP-1, and induction of the ER stress pathway. Additionally, (+)-bornyl *p*-coumarate induced autophagy in both melanoma cell lines, by up-regulating autophagy-related proteins, such as Beclin-1, LC3-I, LC3-II, Atg5, Atg3 and p62 [79].

#### 2.2.4. Other Monoterpenoids

Camphene is a bicyclic monoterpene commonly found in the essential oils of conifers, but also present in other plants (e.g., hemp, rosemary, pepper and nutmeg) [80,81,82,83]. This bioactive compound has been found to have hypolipidemic and weak antinociceptive properties. It also acted as a spasmolytic agent in an experimentally induced bronchospasms in animals [80]. The anti-cancer properties of camphene were studied by Girola et al. on B16F10-Nex2 murine melanoma cell line and in vivo, in a syngeneic mouse model. The authors observed the cytotoxic effect of camphene in B16F10-Nex2 cells, with the IC_50_ of 71.2 μg/mL. The camphene-treated cells showed apoptotic features, such as DNA condensation (pyknotic nuclei) and fragmentation, increased expression of the cleaved caspase 3, as well as phosphatidylserine exposure on the outer leaflet of the plasma membrane. The dose of 70 μg/mL of camphene was shown to elicit the ER-stress-related cell events, such as the elevation of cytosolic Ca^2+^ levels and increased expression of calreticulin and high mobility group box protein 1 (HMGB1). Additionally, the loss of the mitochondrial transmembrane potential was observed, indicating the mitotoxic effect of camphene. Finally, a 20-day treatment of melanoma-bearing mice with daily doses of 10 μg/mL of camphene resulted in a significant decrease in tumour volume [83].

Citral, an aliphatic aldehyde belonging to the monoterpene family, is an isomeric mixture of geranial and neral and is a major component of essential oils of lemongrass (*Cymbopogon citratus*), lemon balm (*Melissa officinalis*), and verbena (*Verbena officinalis*) [84,85,86]. Its antimicrobial or antiparasitic properties have been well described [87,88]. The anti-cancer activity of citral has been reported in experiments on human breast cancer and rhabdomyosarcoma cell lines [89,90]. Sanches et al. conducted a study of the effect of citral on mouse (B16F10), and human (SK-MEL-147 (NRas mutant), UACC-257 (Braf mutant)) melanoma cell lines, as well as on noncancerous cells. The reduction in cell proliferation, as well as decreased cell viability were already observed at 0.5 μM citral, with the IC_50_ value estimated at 1.04 μM (B16F10), 11.7 μM (SK-MEL-147), and 13.4 μM (UACC-257) for a 24 h treatment. The authors linked the diminished cell proliferation to the citral-evoked reduction in the translocation of NF-κB to the nucleus and subsequent decrease in NO levels. The ability of citral to interfere with cell survival-related signaling pathways was proven by revealing the lack of nuclear translocation of the extracellular signal-regulated kinase 1/2 (ERK1/2) and reduced expression of phosphatidylinositol 3-kinase (PI3K) and Akt in the citral-treated melanoma cells. The cytotoxic effect of citral, observed as the induction of apoptosis, autophagy and necrosis, was explained by the authors as the result of citral-generated oxidative stress. Interestingly, the cytotoxic and antiproliferative effects were also registered in non-neoplastic cells, at citral concentration of 50.3 μM in human keratinocytes (HaCaT), and at 2.50 μM in murine fibroblasts (NIH-3T3) [91].

Limonene is an ubiquitous monoterpene, which can be found in *Citrus* plants and pine trees. It is also one of the most abundant terpenes in hemp. It was proved to have antibacterial, anti-inflammatory and anticancer properties [92,93]. The latter were studied in breast and colon cancer models [80,92]. The anti-melanoma effect of limonene was investigated by Alipanah et al. on A375 cell line. The authors assessed the ability of limonene and limonene-containing chitosan nanoparticles to reduce cell viability and compared the results obtained with the cytotoxic properties of limonene-rich essential oils (EOs) and their nanoformulations (NFs). The established IC_50_ value for the pure limonene was more than eight times higher than for the limonene-containing NFs (246.05 vs. 30.24 μg/mL, respectively), suggesting the improved anti-cancer effect of the latter. Interestingly, the cytotoxic effect of all three studied limonene-rich EOs and two out of three investigated EO-nanoparticles was higher than that of pure limonene [94].

Linalool, a terpene alcohol, refers to two enantiomers present in large amounts in essential oils of plants belonging to *Lamiaceae* (e.g., lavender, basil), *Lauraceae* (e.g., cinnamon) and *Rutaceae* (e.g., citrus fruits) families, as well as in many other plant species [80,95]. It is known for its sedative, antidepressant, anxiolytic, analgesic, anticonvulsant, and immune potentiating effects [80]. Its anti-cancer properties were investigated in an ovarian cancer model and in a hepatocellular carcinoma cell line [96,97]. Cerchiara et al. studied the effect of two different ethanolic solutions of linalool (5.60 and 0.56 μM) on the RPMI 7932 human melanoma cell line. A dose-dependent antiproliferative effect of linalool was registered in the melanoma cells. The apoptosis-associated ultrastructural changes in a form of chromatin condensation, nuclear fragmentation, a large number of cytoplasmic vesicles, as well as surface blebbing were observed in melanoma cells by means of transmission and scanning electron microscopy, after the treatment with linalool solutions. The linalool-induced apoptosis was further confirmed by the detection of enhanced expression of caspase 3 in linalool-treated RPMI 7932 cells. Of note, the same treatment evoked little to no changes in the normal human keratinocytes (NCTC 2544 cell line) suggesting only slight toxicity of linalool to non-cancerous cells [98].

### 2.3. Sesquiterpenoids

#### 2.3.1. β-Elemene

β-Elemene (1-methyl-1-vinyl-2,4-diisopropenyl-cyclohexane) (Figure 2), the major component of *Curcuma wenyujin*, a Chinese medicinal plant, has long been studied for its anti-cancer properties. Three carbon–carbon double bonds have been identified as the most important pharmacophores for the anti-cancer activity of β-elemene [99]. Among the various neoplastic models in which the anti-tumour activity of this sesquiterpenoid has been confirmed, there are: human glioblastoma, astrocytoma, colorectal carcinoma, colon carcinoma, pulmonary adenosquamous carcinoma, small-cell lung carcinoma, epitheloid cervix carcinoma, cervical carcinoma, breast adenocarcinoma, and ductal breast epithelial tumour [100,101]. Human melanoma cell line, A375, was used by Balavandi et al. to test the cytotoxic and pro-apoptotic properties of β-elemene. The IC_50_ values calculated for β-elemene were ca. 112.2, 88.43, and 42.06 μg/mL, at 24, 48, and 72 h, respectively. A 24 h pretreatment of the cells with β-elemene (40 and 80 μg/mL), followed by exposure to radiation at doses of 2 and 4 Gy, even stronger reduced cell proliferation than the treatment with the terpenoid alone. The combined therapy also proved to be more effective in inducing apoptosis then the β-elemene or radiation alone, confirming the radio-sensitizing properties of the sesquiterpenoid [102].

Similarly, reduced viability in melanoma cells (B16F10) after the treatment with β-elemene was observed by Shi et al. In that case, the calculated IC_50_ values for β-elemene were approximately 536.1, 447.6, and 332.4 μmol/L, at 24, 48, and 72 h, respectively. Additionally, 40 and 80 μmol/L β-elemene significantly inhibited cell migration, invasion, as well as the expression of metastasis-related proteins, such as urokinase-type plasminogen activator (uPA), uPA receptor (uPAR), MMP-2, and MMP-9, at 24 h [103].

#### 2.3.2. Other Sesquiterpenoids

β-Caryophyllene (BCP) is a sesquiterpene found in various plants, including cloves, black pepper, cinnamon, oregano, thyme, or cannabis. In cancer studies, BCP has been reported to act synergistically with paclitaxel, stimulate apoptosis and inhibit tumour growth [104,105]. Jung et al. studied the effects of BCP on the tumour growth and metastasis of B16F10 melanoma cells in high-fat diet-induced (HFD) obese C57BL/6N mice, as well as in cell cultures, including B16F10, MA (mature adipocytes), M2-Mφ (macrophages), lymphatic endothelial cells (LEC), 3T3-L1 (mouse embryonic fibroblasts) differentiated into preadipocytes, and a coculture of B16F10, MA, and M2-Mφ. The authors proved that dietary BCP (0.3%) suppressed tumour growth and lymph node (LN) metastasis in B16F10 melanoma-bearing HFD-fed mice, which was associated with the inhibition of cell cycle progression and cell survival, as well as suppression of angiogenesis and lymphangiogenesis in the tumour. Additionally, dietary BCP inhibited the HFD-evoked increase in production of cytokines and accumulation of lipids and M2-Mφ in the tumour and LN-surrounding adipose tissue, thus abolishing the obesity-associated changes in the microenvironment of the tumour and LN. Dietary BCP also reduced the HFD-induced increase in the levels of metastasis-associated CCL19 and CCL21 chemokines in the LN, and CCR7 expression in the tumour. In in vitro tests, BCP (5 μM) directly inhibited the production of monocyte migration—and M2 differentiation-related chemokines (MCP-1 and M-CSF) [106].

Guaianolides are natural sesquiterpene lactones demonstrating a wide range of biological activities, including anticancer and anti-inflammatory effects [107]. Estévez-Sarmiento and collaborators tested the anticancer properties of chlorinated guaianolides chlorohyssopifolin A, B, C, D, and E isolated from *Centaurea hyssopifolia* Vahl, as well as linichlorin A and C isolated from *Centaurea linifolia* Vahl on SK-MEL-1 melanoma cells. The authors observed that chlorohyssopifolin A and linichlorin A exerted the most potent cytotoxic effect on SK-MEL-1 cells, with IC_50_ of ca. 3.4 μM and 3.6 μM (72 h), respectively. Of note, chlorohyssopifolin C and D also showed IC_50_ values below 10 μM [108].

### 2.4. Diterpenoids

#### 2.4.1. Andrographolide

Andrographolide (Andro) (Figure 2), a diterpenoid lactone, is the main bioactive constituent of *Andrographis paniculata*, which possesses antibacterial, antiviral, anti-inflammatory and anti-cancer properties. It also has been considered as a promising molecule in developing anti-diabetic drugs [109,110]. The intact γ-butyrolactone ring, a hydroxyl group at C14, as well as double bonds between C12 and C13, and between C8 and C17 have been shown to be mainly responsible for Andro’s cytotoxicity [111]. Zhang et al. studied the effect of Andro in B16 mouse melanoma cells, as well as mouse lung metastasis and subcutaneous melanoma models. They proved that Andro (5 μg/g) not only significantly reduced the tumour volume, but also decreased the number of lung metastatic foci and the size of micrometastatic foci in B16-bearing C57BL/6J mice. In in vitro experiments, Andro (10 μmol/L) was shown to halt the cell cycle progression and induced apoptosis in B16 cell line. Finally, the authors demonstrated that Andro decreased the expression of some representative molecules in a toll-like receptor (TLR)-4 pathway (TLR4, MyD88, pIκBα, pp65, pp50) both in tumour tissue and in cell culture, suggesting that signaling via TLR4/NFκB is the dominant target of Andro in the regulation of melanoma tumorigenesis [112].

The cytotoxic and proapoptotic effects of Andro were investigated in human melanoma cell lines by Liu and Chu. The calculated IC_50_ values were 23.08 (24 h) and 12.07 μM (48 h) for A375 cell line, and 20.31 (24 h) and 10.92 μM (48 h) for C8161 cells. The authors reported a dose-dependent cell cycle G2/M arrest in both studied cell lines and induction of apoptosis observed as enhanced expression of cleaved caspase 3 and PARP in A375 cells, already after a 24 h treatment with 5 μM Andro. The upstream events associated with cell cycle arrest and apoptosis induction were proved to be related to phosphorylation and activation of JNK and p38 [113].

#### 2.4.2. Other Diterpenoids

Carnosic acid (CA), also known as salvin, is found in high amounts in the extracts from *Salvia* and *Rosmarinus*. Its antioxidant and antimicrobial properties have long been studied, in addition to the anti-cancer, chemoprotective and anti-inflammatory properties of CA-rich plant extracts [114]. The anti-melanoma activity of CA was explored by Park et al. in B16F10 cells. The authors proved that CA (2.5–10 μM) dose-dependently inhibited the migration of melanoma cells, which suggested the suppression of epithelial-to-mesenchymal transition (EMT)-related mechanisms. This assumption was confirmed by the observed reduction in the secretion of proteases associated with cancer invasion and metastasis (MMP-9, uPA), and an increase in the level of tissue inhibitor of metalloproteinase (TIMP)-1 in cells treated with 10 μM CA. Additionally, it was noticed that CA at 5 or 10 μmol/L suppressed EMT by reducing the expression of mesenchymal markers (vimentin and N-cadherin), EMT-associated transcription factors (Snail and Slug), and inhibition of the phosphorylation of Akt and Src/FAK signaling pathway components [115].

Triptolide is a bioactive constituent of a Chinese medicinal plant *Tripterygium wilfordii*, known for its immunomodulatory effect. Its anti-cancer effects have been studied, among others, in breast and colorectal cancer, as well as in melanoma [116,117,118,119]. As reported by Jao and collaborators, triptolide significantly inhibited cell-matrix adhesion (20 nM in 24 h, 10 and 20 nM in 48 h), and suppressed cell migration (starting from 10 nM in 24 h) and invasion (starting from 5 nM, in 24 h) in B16F10 cell melanoma model. The observed mechanisms underlying this anti-cancer effect of triptolide included downregulation of receptors (CXCR4), kinases (p-ERK, FAK, p-Akt, p-JNK), adapter proteins and other protein regulators (GRB2, SOS1, Rho A, ROCK-1), transcription factors (NF-κB), and effector proteins (MMP-9, MMP-2) that are involved in the promotion of cell migration and invasion [120].

One of the tetrahydroingenol diterpenoids isolated from *Euphorbia erythradenia* is 7,13-Diacetyl-5-angeloyl-20-nicotinyl-3-propionyl-1,2,6,7-tetrahydroingenol (DANPT). Its strong anti-proliferative activity has been demonstrated in experiments on human ovarian and bladder carcinoma cell lines [121]. Significant cytotoxicity of DANPT towards human melanoma cells (A375, HMCB), with the IC_50_ value of ca. 15.37 μM (A375) and 15.62 μM (HMCB) after a 48 h incubation, was shown by Fallahian et al. As proved by the authors, this cytotoxic effect was due to the induction of oxidative stress, which evoked the p53 activation, and subsequently led to cell cycle arrest in G2/M phase and apoptosis [122].

### 2.5. Triterpenoids

#### 2.5.1. Ursolic and Oleanolic Acid

Natural pentacyclic triterpenoids, ursolic acid (UA) and its isomer—oleanolic acid (OA) (Figure 2) are especially abundant in apple peel, bilberries, cranberries, elder flower, lavender, oregano and thyme. Their pro-health properties include the antihyperlipidemic, antidiabetic, hepatoprotective, antibacterial, antiviral, antifungal, anti-inflammatory, antioxidant, and anti-cancer action [35,123,124,125,126]. As has been demonstrated, the number and positions of hydroxyl groups attached to the terpene structure affect the activity and selectivity of ursane-type pentacyclic triterpenoids for cancer cell lines [127]. Additionally, C-3, where a hydroxyl group is attached, C-28 (a carboxylic group), and a double bond between C-12 and C-13 can serve as the main sites of modifications which enhance anti-cancer activity of ursolic and oleanolic acid [128,129]. As reported by Lee et al., pretreatment with UA potentiated UVR-induced cytotoxic effects in skin melanoma cells (CRL-11147 cell line). Ursolic acid greatly intensified UVR-evoked oxidative stress and collapse of mitochondrial membrane potential, and, consequently, enhanced G1 phase arrest and apoptosis. Interestingly, the contrary effects were observed in UA-pretreated, UVR-exposed non-cancerous, retinal pigment epithelium cells (CRL-4000 hTERT-RPE cell line), suggesting a photoprotective effect of UA on healthy cells [130].

The results obtained by Caunii et al. in the experiments on SK-MEL-2 confirmed the cytotoxic effects of UA and allowed to calculate the IC_50_ value, which was approximately 58.43 μM, for a 48 h incubation. The effects were observed as induction of the cell cycle arrest in the S phase and reduction in cell adhesion capacity. When compared to UA, OA showed lower cytotoxic effects in the in vitro tests. However, it exerted stronger inhibitory effect on the invasive and angiogenic properties of melanoma, in an in vivo chicken chorioallantoic membrane assay (CAM) [131].

#### 2.5.2. Other Triterpenoids

Cucurbitacins (Cucs) are a group of highly oxygenated tetracyclic triterpenoids, naturally occurring in the plants from *Cucurbitaceae* family, such as pumpkin, cucumber, or melon. As a part of the plants’ defense mechanism, they possess the antimicrobial, antiviral, and anti-inflammatory properties. Their anti-tumour effects were proven in cervical, lung, colon, bladder and prostate cancer, as well as in hepatoblastoma-derived cell lines [132,133,134,135]. In the study conducted by Ahmed and Halaweish, Cucs exhibited cytotoxicity on the SK-MEL-28 and A375 cell line, with the lowest IC_50_ value of ca. 0.32 μM, for Cuc D (A375), and 0.36 μM for Cuc B (SK-MEL-28). The authors demonstrated that Cucs have a strong binding affinity towards the crystal structures of RAF and MEK, which surpassed that of a MEK inhibitor, PD0325901, in case of Cuc B and Cuc E. Additionally, Cucs revealed their inhibitory potential towards the total and phosphorylated ERK, suggesting blocking of the MAP kinase signaling pathway (A375) [136].

Betulinic acid (BA) is a pentacyclic lupane-type triterpenoid with confirmed anticancer properties. Its cytotoxic effect has been observed in many different cancer cell lines, including the in vitro models of glioblastoma, neuroblastoma, melanoma, and lung and cervix carcinoma, among others [137]. A dose-dependent decrease in cell viability, with IC_50_ of 16.81 μM (24 h), was reported by Coricovac et al. in A375 cell line treated with BA. Furthermore, the cells subjected to 10 and 50 μM concentrations of BA, for 24 h, expressed morphological and molecular features characteristic of apoptosis, such as nuclear fragmentation, and up-regulation of proapoptotic marker genes (Bax, Bad, and Bak), respectively. BA-evoked mitochondrial dysfunction was observed as mitochondria depolarization, a decrease in the oxygen consumption rate (OCR) and extracellular acidification rate (ECAR), a significant decrease in oxidative phosphorylation (OXPHOS), and in the maximal respiratory capacity of the electron transport system (ETS). Molecular features of mitochondria dysfunction were accompanied by distribution of mitochondria at the periphery of the nucleus and reorganization of actin microfilaments. Of note, a dose-dependent decrease in cell viability, as well as morphologic features of apoptosis were also observed in noncancerous immortalized human keratinocytes (HaCaT cell line), but at higher BA concentrations (20 μM and higher) [138].

**Figure 3:**
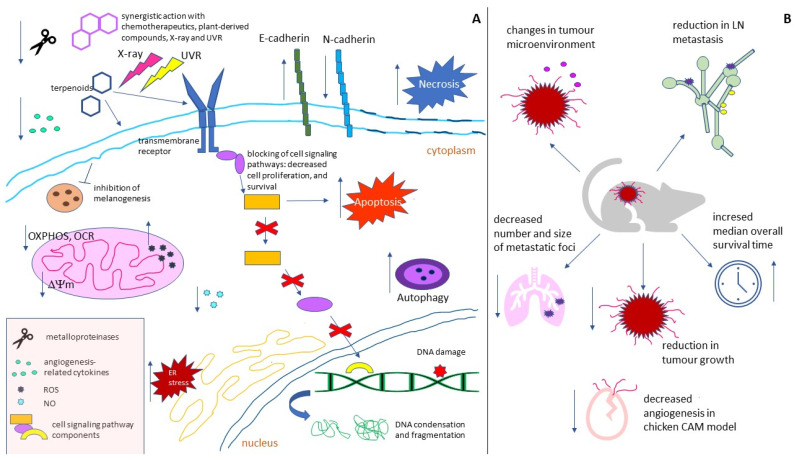
The action mechanisms of plant-derived terpenoids in (**A**) melanoma cell lines, and (**B**) in vivo melanoma models and chicken CAM. (**A**) Terpenoids were found to exert a cytotoxic effect alone or in combination with other plant-derived bioactive compounds and chemotherapeutics/radiation. On the molecular level, they interfere with cell signaling pathways; block melanogenesis; and induce mitochondria dysfunction, ER stress, and DNA damage, which subsequently leads to apoptosis, autophagy, or necrosis. Terpenoid-caused decrease in expression/secretion/activity of EMT- and angiogenesis-associated molecules results in reduction in EMT, migration, and invasion. (**B**) In animal models, terpenoids were shown to induce changes in tumour and lymph node microenvironments, and reduce angiogenesis and LN metastasis that result reduced tumour mass, and number and the size of metastatic foci, and increased survival time of the tumour-bearing animals.

The effects of the treatment of melanoma cells and tumour-bearing animals with plant terpenoids are summarized in Table 1 and in Figure 3.

## 3. Conclusions

Despite the large number of therapeutic approaches for treating malignant melanoma, the cutaneous form of this cancer remains responsible for 0.6% of cancer-caused deaths worldwide. Various genetic changes underlying the initiation and progress of melanoma, as well as the wide range of its defense mechanisms, necessitate the constant search for new strategies in the fight against this cancer. Natural terpenoids have recently been gaining more and more attention as potential melanoma-targeting therapeutics, or the agents sensitizing melanoma cells to standard forms of therapy. Their undoubted advantage is their lower cytotoxicity compared to classic chemotherapeutic agents. Research from the last eight years show that in in vitro melanoma models, plant terpenoids: (1) demonstrate dose-dependent cytotoxicity; (2) induce apoptosis, necrosis, or autophagy; (3) evoke increased generation of ROS, oxidative stress, and loss of ∆ψm; (4) decrease OCR, ECAR, OXPHOS and the maximal respiratory capacity of ETS; (5) induce ER stress; (6) cause cell cycle arrest; (7) induce DNA damage; (8) decrease the expression and lower the activity of proteins involved in melanogenesis; (9) interfere with the cell-signaling pathways responsible for cell growth, proliferation, migration, adhesion, and invasion; (10) decrease the expression of angiogenesis-related cytokines; (11) inhibit EMT; (12) cause radio- and photosensitization; and (13) act synergistically with other natural compounds, or chemotherapeutics. 

In in vivo melanoma models, terpenoids (1) increase the median overall survival time of tumour-bearing animals; (2) cause a reduction in tumour volume; (3) decrease the expression of metastasis-associated chemokines and receptors, as well as lymph node metastasis; (4) decrease the number and size of metastatic foci; (5) change the tumour microenvironment and the lymph node-surrounding adipose tissue; and (6) inhibit angiogenesis. At the same time plant-derived terpenoids express generally lower-to-no toxicity to non-cancerous cells, or increase their photoprotection.

Plant terpenoids are a group of promising therapeutic/adjuvant substances for treating melanoma. They are, though, being poorly soluble in an aqueous solution, but this problem is being successfully overcome by the use of nanoparticles as a drug delivery system. 

## Figures and Tables

**Table 1:** The effects of natural plant terpenoids in in vitro and in vivo melanoma models.

Studied Effect	Terpenoid/Cell Line or Melanoma In Vivo Model	Reference
Dose-dependent cytotoxicity	TQ (B16F10); TQ-PLGA NPs (A375); TQ + Iksan526 (A375, B16F10); α-terpineol in PMMA nanoparticles (B16F10, SK-MEL-28); terpinene-4-ol alone, or in combination with dabrafenib, or trametinib (M14, A375); NB + curcumin (A375); bornyl *cis*-4-hydroxycinnamate (A2058, A375); bornyl *p*-coumarate (A2058. A375); camphene (B16F10-Nex2); citral (B16F10, SK-MEL-147, UACC-257); limonene, limonene-containing nanoparticles, limonene-enriched EOs, and their NFs (A375); linalool (RPMI 7932); β-elemene alone, or in combination with X-ray (A375); β-elemene (B16F10); chlorinated guaianolides (SK-MEL-1); Andro (B16, A375, C8161); triptolide (B16F10); DANPT (A375, HMCB); UA alone, or in combination with UVR (CRL-11147); UA and OA (SK-MEL-2); Cucs (SK-MEL-28, A375); BA (A375)	[57,59,61,62,68,69,74,77,78,79,83,91,94,98,102,103,108,113,120,122,130,131,136,138]
DNA damage	TQ (B16F10)	[57]
morphological features of apoptosis/phosphatidylserine translocation	TQ (B16F10); NB + curcumin (A375); bornyl *cis*-4-hydroxycinnamate (A2058, A375); bornyl *p*-coumarate (A2058. A375); camphene (B16F10-Nex2), citral (B16F10); linalool (RPMI 7932); β-elemene (A375); Andro (B16); DANPT (A375, HMCB); UA alone, or in combination with UVR (CRL-11147); BA (A375)	[57,58,74,77,78,79,83,91,98,102,112,122,130,138]
increased ROS generation/oxidative stress	TQ (B16F10); NB + curcumin (A375); citral (B16F10), DANPT (A375, HMCB); UA + UVR (CRL-11147)	[57,74,91,122,130]
mitochondria loss of function/loss of ∆ψ m	bornyl *cis*-4-hydroxycinnamate (A2058, A375); bornyl *p*-coumarate (A2058, A375); camphene (B16F10-Nex2); UA + UVR (CRL-11147); BA (A375)	[77,79,83,130,138]
decrease in OCR	BA (A375)	[138]
decrease in ECAR	BA (A375)	[138]
decrease in OXPHOS	BA (A375)	[138]
decrease in maximal respiratory capacity of ETS	BA (A375)	[138]
ER stress	bornyl *cis*-4-hydroxycinnamate (A2058, A375); bornyl *p*-coumarate (A2058, A375); camphene (B16F10-Nex2)	[77,79,83]
cell cycle arrest	BCP {HFD-induced obese C57BL/6J mice with B16F10}; Andro (C8161, A375, B16); Andro (A375); DANPT (A375, HMCB); UA + UVR (CRL-11147); UA (SK-MEL-2)	[106,112,113,122,130,131]
necrosis	citral (B16F10)	[91]
autophagy	bornyl *p*-coumarate (A2058, A375); citral (B16F10)	[79,91]
increased caspases/cleaved caspases 3/8/9	TQ (B16F10); terpinene-4-ol alone, or in combination with dabrafenib, or trametinib (M14, A375); NB + curcumin (A375); bornyl *cis*-4-hydroxycinnamate (A2058, A375); bornyl *p*-coumarate (A2058, A375); camphene (B16F10-Nex2); linalool (RPMI 7932); Andro (A375)	[57,69,74,77,79,83,98,113]
increased PARP/cleaved PARP	terpinene-4-ol alone, or in combination with dabrafenib, or trametinib (M14, A375); NB + curcumin (A375); bornyl *cis*-4-hydroxycinnamate (A2058, A375); bornyl *p*-coumarate (A2058, A375); Andro (A375)	[56,74,77,79,113]
decreased anti-apoptotic proteins/genes (Blc-2, Bcl-xl, Mcl-1)	TQ (B16F10); bornyl *cis*-4-hydroxycinnamate (A2058, A375)	[57,77]
increased proapoptotic proteins/genes (Bax, Bad, Bak)	TQ (B16F10); bornyl *cis*-4-hydroxycinnamate (A2058, A375); BA (A375)	[57,77,138]
decreased survivin	TQ (B16F10)	[57]
increased cytosolic cytochrome c	bornyl *cis*-4-hydroxycinnamate (A2058, A375)	[77]
increased p53	NB + curcumin (A375); DANPT (A375, HMCB)	[74,122]
decreased regulators and participants of melanogenesis (MITF, tyrosinase)	TQ + Iksan526 (A375, B16F10)	[62]
decreased tyrosinase activity	TQ (B16F10)	[59]
decreased COX-2	TQ + Iksan526 (A375, B16F10)	[62]
decreased expression of NF-κB/lack of its nuclear translocation and DNA binding	citral (B16F10); triptolide (B16F10)	[91,120]
blocking of JAK2/STAT3	TQ (B16F10)	[57]
ERK 1/2 pathway inhibition	NB + curcumin (A375); citral (B16F10); triptolide (B16F10); Cucs (A375)	[74,82,120,136]
decreased expression of FAK/PI3K/Akt/mTOR pathway proteins, or their phosphorylated forms	NB + curcumin (A375); bornyl *cis*-4-hydroxycinnamate (A2058, A375); citral (B16F10); CA (B16F10); triptolide (B16F10)	[74,78,91,115,120]
increased p-JNK	NB + curcumin (A375); Andro (A375)	[74,113]
reduced p-JNK	triptolide (B16F10)	[120]
increased p-p38	Andro (A375)	[113]
decreased p-Src	CA (B16F10)	[115]
decreased β-catenin	TQ (B16F10); CA (B16F10)	[59,115]
decreased GRB2 pathway members	bornyl *cis*-4-hydroxycinnamate (A2058, A375); triptolide (B16F10)	[78,120]
decreased CXCR4	triptolide (B16F10)	[120]
decreased SOS 1	triptolide (B16F10)	[120]
decreased Rho A	triptolide (B16F10)	[120]
decreased Rock-1	triptolide (B16F10)	[120]
decreased NO	citral (B16F10)	[91]
increased p-Brca1 and p-ATM	NB + curcumin (A375)	[74]
decreased expression of angiogenesis-related proteins (MCP-1, TGF-β1, RANTES)	TQ (B16F10); BCP (B16F10)	[59,106]
inhibition of angiogenesis	BCP {HFD-induced obese C57BL/6J mice with B16F10}; OA {chicken CAM}	[106,131]
decreased lymphangiogenesis	BCP {HFD-induced obese C57BL/6J mice with B16F10}	[106]
decreased M-CSF	BCP (B16F10)	[106]
decreased TLR 4 pathway components	Andro (B16); Andro {C57BL/6J mice with B16 melanoma subcutaneous model}	[112]
decreased cell-matrix adhesion	triptolide (B16F10); UA (SK-MEL-2)	[120,131]
decreased expression/secretion of uPA and uPA receptor	β-elemene (B16F10); CA (B16F10)	[103,115]
reduced CCL19 and CCL21 in LN	BCP {HFD-induced obese C57BL/6J mice with B16F10}	[106]
reduced CCR7 in tumour	BCP {HFD-induced obese C57BL/6J mice with B16F10}	[106]
increased TIMP-1	CA (B16F10)	[115]
decreased EMT/EMT-associated proteins (vimentin, N-cadherin, Snail, Slug)	bornyl *cis*-4-hydroxycinnamate (A2058, A375); CA (B16F10)	[78,115]
increased E-cadherin	bornyl *cis*-4-hydroxycinnamate (A2058, A375)	[78]
decreased activity/expression/secretion of MMP-2/MMP-9	bornyl *cis*-4-hydroxycinnamate (A2058, A375); β-elemene (B16F10); CA (B16F10); triptolide (B16F10)	[78,103,115,120]
decreased cell migration/invasion	bornyl *cis*-4-hydroxycinnamate (A2058, A375); β-elemene (B16F10); CA (B16F10); triptolide (B16F10); OA (SK-MEL-2)	[78,103,115,120,131]
reduced LN metastasis	BCP {HFD-induced obese C57BL/6J mice with B16F10}	[106]
increased median overall survival time of tumour-bearing mice	TQ {C57BL/6J mice with B16F10}; TQ + gamma knife {C57BL/6J mice with B16F10}	[58,59]
reduction in tumour growth	camphene {C57BL/6J mice with B16F10}; BCP {HFD-induced obese C57BL/6J mice with B16F10}; Andro {C57BL/6J mice with B16 melanoma subcutaneous model}	[83,106,112]
changes in tumour microenvironment and LN-surrounding adipose tissue	BCP {HFD-induced obese C57BL/6J mice with B16F10}	[106]
decreased number and size of metastatic foci	Andro {C57BL/6J mice with B16 melanoma lung metastasis model}	[112]
radio-sensitization	β-elemene (B16F10)	[102]
phototoxicity	UA + UVR (CRL-11147)	[130]
synergistic action with chemotherapeutics	terpinene-4-ol in combination with dabrafenib, or trametinib (M14, A375)	[69]

Round and curly brackets were used to indicate in vitro (cell lines), and in vivo melanoma models, respectively.
